# COVID-19, Social Policy and Care: A Complex Set of Processes and Outcomes

**DOI:** 10.3389/fsoc.2021.808239

**Published:** 2022-01-20

**Authors:** Mary Daly

**Affiliations:** Department of Social Policy and Intervention, University of Oxford, Oxford, United Kingdom

**Keywords:** care, social policy, childcare, long-term care, European welfare states, European care systems

## Abstract

This article looks at the 2020 period of COVID-19 and especially the first months through the lens of public policy support for care in Europe. It covers the policy responses to both care for young children and frail, ill or disabled adults and develops an understanding of care as welfare-related activity focused on practices and resources oriented to meeting care-related need. The article’s over-arching research question centres around how European countries responded to the 2020 pandemic, especially in regard to the types of care need that were recognized, the resources committed, the actors/agency that were supported or taken for granted and the values underpinning the responses. What we find from the review is that, while care assumed a strong place in public rhetoric, this was not reflected in greater public resourcing of care for young children or long-term care. Instead, care for children was refamilialized and long-term care was under-resourced and relegated to a secondary position; both were in many ways rendered further dependent on the private agency of individuals. In sum, the pandemic spearheaded some reversion to old practices and the opportunity to invest in care as a human need, a basis of rights and entitlements and a valued activity was not availed of.

## Introduction

COVID-19 is a crisis of care. As the pandemic has moved around the world, it has been represented mainly as a medical crisis and phenomenon, to be combatted by specialist medical treatment including drugs and intensive care, and mass medical mobilization through vaccination. While there is of course truth to this, the evolution of the pandemic points the dial strongly towards non-medical care as well. Think of the phenomenon of “long COVID” which might affect up to a fifth of those infected. Consider that, with vaccination, the virus is less likely to require medical intervention and more likely to call for the type of care associated with a low-grade illness such as a flu. Bring to mind the heightened impact of existing vulnerability and how closely it intersects with the likelihood of being infected and affected by the virus prevention and recovery measures. Not just does care-related vulnerability heighten the likely risk but care-related resources and provision play a large role in potentially minimizing exposure and aiding recovery should one contract the virus. All of this calls for a broader view.

This paper looks at COVID-19 through the lens of public policy on care. What does this mean? By care we refer not so much to care in a medical setting or for illness but rather to what one might understand as “everyday care need and related activity”, especially for those who cannot fully take care of themselves. Young children and frail, ill or disabled adults come immediately to mind. But care is a much wider phenomenon than caring for those who have a care need associated with illness, disability, and immaturity or dependency. Sevenhuijsen (2003): 181 speaks of care as “the maintenance of body, soul and relationships that lies at the heart of good human functioning”. This view of care derives from a perception of vulnerability as universal and the human condition as one of interdependence. The concept of care, therefore, embodies a broader condition and philosophy of engagement around well-being, stretching from intimates to strangers and across spheres from home and family to community, society, and the global world. For the purposes of this article I take the narrower definition of care, conceiving of it as welfare-related activity focused on practices and resources oriented to meeting care-related need. That said, the broader more universal understanding of care permeates the piece as a whole.

Encapsulating the state’s response, policy analysis is at the article’s epicenter, examined for what it reveals about support for the practices and needs associated with care as well as the broader web of social, political, and economic institutions in which caring is located (Urban 2020: 283). In short, I view policy as a major contributor to the “care system” in societies. The kinds of policies that care analyses encompass include payments and subsidies allocated to caregivers or to people who need care, direct provision of care services and labour regulations, such as maternity protection and paternity leave and the regulation of paid working times, which assign time from employment for care. Care policies, therefore, encompass policies developed by various sectors, including health and education, labour, and social protection (Esquivel 2017). In its analysis of social policy, the article covers developments in care for both adults (the general field of long-term care) and children (especially that for young children in the field of early education and child care). This perspective is adopted for several reasons, not least because it allows a more inclusive understanding of care and makes for a more integrated policy analysis, connecting fields that are typically separated and truncated into “old age” and “early life course”.

The over-arching question underlying this article centres on how public policy has approached care need and provision during the pandemic, especially in regard to the types of care need that have been recognized, the resources committed, and the relationships and locations of care that are supported. The overall analysis aims to lay bare the key priorities, relationships and resources that are contained in how countries in Europe responded and to assess the extent to which the pandemic led to a change in approach, although a “health warning” is apposite here as it is too early to draw definitive conclusions about a pandemic that is still evolving. Because of evidence shortages which in turn force reliance on information that is still incomplete, the comparisons undertaken are broad-brush in nature and cannot always give equivalent attention to long-term care and care for children.

The paper is in three main parts. The first offers some thoughts on a framework for understanding and analysing care in the COVID-19 context. The second part undertakes a review of policy’s interpretation of care need, the resources committed, the actors and care-related agency that were supported and the underlying value set. A short concluding section follows which sets out especially some of the main alternative models or discourses on the organization of care and how it might be better valued.

## Framework of Analysis/Orientation

To begin, some words are necessary about how to conceptualize and study COVID-19. There is no consensus in the literature on how we should configure the pandemic: for some it is a short-term event, requiring an emergency response; for others it is a critical juncture for health systems but also for the welfare state more broadly. This article tends towards the latter view, seeing the pandemic as revelatory of both short-term tendencies and long-term, underlying patterns around how societies organize to obtain human welfare and well-being. That said, there are real difficulties in studying pandemic policy. These include its changing and fast-moving constitution and associated transformations in nature, timing, extent and impact within and across countries. There is also the fact that the pandemic has necessitated an emergency response from systems that generally evolve only slowly. All of this is reflected in the changing policy response; with the pandemic seemingly in a third or fourth wave we are seeing mark two or three of some original policy responses (although it is not clear that there is much that is actually new given that later policies appear to be an iteration or winding up or down of initial policies with some lessons learnt). When this general scenario is put together with continuing information shortages about what actually has happened or is happening, we find ourselves theorizing and researching in a rather “small space”. I am compelled to make two concessions in light of this. First, the main focus of this article is on the first period of the pandemic in Europe (from onset in February/March 2020 to late Summer 2020) given that it is that period on which most data and research are available. Second, I also have to scale back the paper’s purpose and would underline therefore that the aim is as much to set out a framework and lay the groundwork for understanding policy responses during the pandemic to help future scholarship as to provide definitive answers to big questions at this stage.

In organizing and considering the evidence, I utilize a framework that I developed in an earlier paper (Daly 2021) to theorize policies on care provision (rather than, say, broader political mobilizations around care). In it, I suggested that perceived need—and especially welfare- and/or personal support-related need—and the response to it might be thought of as at the core of a policy-oriented conceptualization of care. My thinking here underlines that policy always entails an interpretive process in adjudicating on what is a legitimate need and therefore meriting recognition and resourcing. This approach suggests that any framework must comprehend relations of hierarchy and relative powerlessness and, indeed, the political nature of need definition and adjudication (Fraser 1989). Developing this type of understanding of perceived need and the responses to it, the other component elements of my framework are relations and actors, resources, and ideas/values.

“Relations” signify care as a set of relational practices connecting all the interactions involved in providing and/or receiving care. Focusing on relations moves the spotlight away from both individualized conceptions of care and dichotomous thinking to emphasize processes and ecological embeddedness. It also avoids the passive register which denies or takes for granted care-related agency. Adding “actors” to the care configurations takes us further in the direction of agency, granting recognition to both the breadth of agency and the range of actors that are or might be involved. Thinking in terms of “resources” compels us to enquire about all the activities that are involved in care and such important matters as whether care is paid or not and the support services and other forms of organization that are put in place, especially by the state or public authorities. The third vector—“ideas and values”—plumbs the ideational and moral framing of care and directs enquiry to such factors as prevailing philosophies and idea(l)s of care and perceptions of the value and legitimacy of “claims” around care.

This framework is used to interpret systematically the different elements. In each case I consider what an alternative response might have been.

## Empirical Developments and Trends

### Interpretations of Need

As mentioned, need and especially social need is not a fact (or not solely a fact) but is, instead, interpreted. This type of thinking derives especially from constructivist approaches and critical gender studies (as in Carol Bacchi (1999) “What's the Problem?). Political philosopher Nancy Fraser wrote about the politics of need interpretation in a 1989 book, underlining the assumptions and subtext of how needs are interpreted, the selectivity involved and the political nature of adjudicating on claims for public resources. One might note that as state resources are reduced (under austerity policies for example) the interpretation of need becomes ever more intensive—the growth of conditionality as a principle governing access to welfare benefits and services is one way in which a narrow and particularistic interpretation of need is generalized (Dwyer 2019).

Looking at what has happened during the pandemic for adults in need of care especially, we can see two processes: a medicalization of need and a hierarchicalization of care-related need. Both draw on long-term tensions and ambivalences in the field of care. In regard to medicalization, the most legitimate or recognized needs have been of people who were ill—they were prioritized for services and resources, especially medical services in a context where many wider health and other services were cut back or withdrawn. This tendency also elevated medical care as the core (and sometimes sole) element of the desired response at the pandemic’s onset, exacerbating tendencies to see social care as the poor relation or residuum of medical care. A related trend was of hierarchicalization. This manifested in a relative downgrading if not neglect of those in need of more everyday or routine care. In consequence, many countries reported high death rates among those in long-term care; on average in the OECD people in residential care accounted for some 41% of all COVID-19-related deaths (up to early February 2021) (Rocard et al., 2021: 16).

The downgrading or special position of long-term care is deeply rooted and manifests in such phenomena as informalization, a lack of information about the care sector within and across countries, under-regulation and relatively poor resourcing (Comas Herrera et al., 2020; Daly et al., 2021). Hierarchicalization was especially to be seen in the downgrading of care homes and long-term care facilities more generally for resources and protection during the pandemic which, while not universal in Europe, was quite common, and especially in the early responses (Daly et al., 2021; Rocard et al., 2021). Provision of care resources for people receiving care at home was also generally cut back, and again without much consideration for those who needed it and/or provided it. Some countries did learn lessons from earlier mistakes when it came to vaccinations however, prioritizing formal, long-term care settings, residents and staff for vaccination. That said, no country has prioritized informal carers for vaccines or other protections. Apart from resources, there is also the question of rights. There is no evidence of expansion of care-related rights either for adults or children during the pandemic and in some settings there was even a diminution—in the United Kingdom for example the right to be resuscitated was withdrawn from care home residents (Daly 2020a; Comas-Herrera et al., 2020). Rottenberg and Segal (2020) characterize the response in the United Kingdom and elsewhere in the early COVID-19 period as one of “care-lessness”.

Explaining these developments is not straightforward. In some respects, they might originate from the over-arching health/medical interpretation of the pandemic. Another possible explanation is of logistical and regulation difficulties in mounting a response in the generally more fragmented, diverse and already less well-resourced adult care sector which, in comparison to the health sector, tends also to be less professionalized and to have poorer working conditions (Addati et al., 2018; Spasova et al., 2018; Rocard et al., 2021). Similarly, it seems that in the field of childcare, policies were ill-prepared for COVID-19, and short-term responses often bridged the way in the absence of longer-term solutions for integrating all children into the “new normal” of early childhood education and care and schooling under pandemic conditions (Blum and Dobrotić 2020). But even if a crisis interpretation holds, the response reveals something quite profound about the underlying social and political consensus and trends over time. For one might well argue that what we are seeing during the pandemic is a continuation of trends established through neo-liberal and austerity policies whereby the protection of and response to vulnerability is no longer considered part of the social contract. The need to act in a hurry notwithstanding, it suggests that priorities lay elsewhere other than with those who have non-medical care need.

What might have happened instead? While not underestimating the great dilemmas and challenges involved, we might well have expected rights to be strengthened and protections put in place regarding the needs of children and adults who require care, many of whom are vulnerable. As regards children, a trend prior to the pandemic was for European countries to increasingly guarantee early childhood education and care as a right for children (Daly 2020b) but the rush to shut down facilities did not generally respect this. Only three countries that I know of kept the childcare facilities open—Finland, Estonia, and Sweden (and the latter two did not operate a general lockdown when the pandemic first hit). A further two—Denmark and the United Kingdom—enabled the early childcare and education facilities to stay open for vulnerable children (defined educationally, in health terms and also socially) (Blum and Dobrotić 2020; Gentilini et al., 2020). Even if new rights were not granted—and here we have to recognize the scale of change and the political capital needed to make something into a right—we might also have expected care provision—both for children and adults—to have been recognized as needing greater support as a vital field in the fight against COVID-19. In relation to long-term care in particular, we might also have expected a better joining up of medical care and non-medical care and a greater recognition of their inalienable linkages, even if they are treated separately for bureaucratic purposes. While a few EU countries (e.g., Estonia, Finland, Latvia, Luxembourg, Portugal and Slovenia) generated guidelines for better integration with hospitals and measures with respect to multidisciplinary teams in long-term care, such approaches were not adopted by the majority of EU or OECD countries. Moreover, only a handful of countries (Greece, Belgium, Estonia, France, Hungary and Netherlands) have introduced new guidelines on the integration of long-term care and care in hospitals (Rocard et al., 2021: 70–73).

### The Actors and Relations

Some suggest that care “came out” during the pandemic. Fine and Tronto (2020: 302) describe this in the sense of it “emerging from the shadows as a taken-for-granted afterthought in public life”. One could find support for this argument—but only, I suggest, if one takes a broad definition and include in care acts of relatively minor but of course important support (such as shopping, help with other chores and so forth) that were at the epicenter of greater solidarity and neighbourliness during the first lockdown periods of the pandemic. These might be conceived as acts of caring citizenship (following Sevenhuijsen 2003) or of “caring about” (one of the four types of caring articulated by Fisher and Tronto (1990)).^1^ Care-related agency of everyday local citizens appears to have flourished, although we should be careful in interpreting the extent and nature of this until more research is available. But even if we accept a groundswell of caring citizenship, rather than coming out care was actually further “interiorized”, within institutions and also in the home and private sphere. Lockdown especially meant that care was not only locked down but also “locked in”—with care institutions largely rendered private and much care confined to home and family. There was massive recourse to privatized actions and actors especially in family and community settings. The relocations of care that Sevenhuijsen (2003) spoke of decades earlier—referring to care moving from women to men and from “inside” to “outside”—were reversed. As a result, we learned little about the reality of the material and affective labour involved in care provision during the pandemic.

Place matters in several respects. One is visibility. The desire by scholars such as Elson (2017) and Folbre (2019) among others to see care recognized is pertinent here—it is hard to confer recognition on something that is private and fully or partially hidden. There is also the matter of transparency—activities located in private or semi-private settings are hard to monitor and difficult to regulate. Location serves to define care in other ways as well. Its primary location in the home or familial setting—including its association with women in the domestic sphere—and the boundaries drawn between it as paid or unpaid work have been vital in shaping the value placed on it and have contributed especially to its relative devaluation and naturalization as women’s work. It is here especially that care has a connection with social reproduction, which theorizes how the maintenance of social life is shaped by and contributes to the ongoing production of an overall capitalist system wherein significant costs for its reproduction are borne by paid and unpaid care and carers (Bakker 2007; Fraser 2016). It is important to note that the shift to home-based care observed during the pandemic is at odds with trends towards multi-locationality of care (Sevenhuijsen 2003). This latest relocation of care is not just a physical phenomenon but also an act with moral and political significance. Family and family members are rendered responsible for care. This has many implications—not least reinforcing an unequal social distribution of care, with much research drawing attention to the gendered nature of care provision during COVID-19 thus far (Power 2020; Fisher and Ryan 2021).

One related striking point is the silencing of those who were in receipt of care (whether at home or through more public channels). These conceived as “recipients” are not accorded much agency anyway but the lack of attention to them as individuals in their own right during the pandemic was notable. Children are an important case in point. Schools were shut down, seriously curtailed or teaching was transferred to an online format in most countries, without seemingly much thought being given to the welfare and education of children (Blum and Dobrotić 2020). Some online resources were made available in some countries as well as food-related provision for children in later months of the first school lockdown (although this depended on whether school meal provision was in place prior to the pandemic). But the primary assumption was that families would take care of children and their learning in the event of withdrawal of the public authorities. Although not identical, generally similar processes served to silence adult care recipients.

What might have happened instead is more resourcing and recognition of the actors and agency involved and greater public responsibility for care. For example, while there was greater public recognition of and rhetorical support for care workers, through phenomena such as *clap for carers,* and the redesignation of them as “key workers”, one might have expected to see recognition expressed in other terms such as greater recompense for care workers through wage increases or stronger benefit and service entitlements. There have been some relevant developments. When recompense was envisaged, the preference seems to have been for one-off pay bonuses for long-term care workers—the OECD reports that about 40% of 20 surveyed countries provided a one-off bonus to reward long-term care workers of their efforts during the first wave of the pandemic (Rocard et al., 2021). In Germany, for example, all people employed in old age care were able to make a staggered claim for a one-off bonus payment of up to €1,500 tax-free. Only a few countries improved wages permanently following the start of the pandemic (Czech Republic, France, and Germany) (Hemmings 2021; Rocard et al., 2021). Again to take Germany as an example, from April 1, 2022, the hourly minimum wage for this profession will be set at €13.20. The OECD study reports that most countries did not implement specific support to informal carers (Rocard et al., 2021: 48).

### Resourcing of Care

Research confirms that care need and care deficits grew during the pandemic (Barry and Jennings 2021). Looking across European countries at the situation of persons aged 50 years and over in home settings who are in need of care or provide informal care to adults outside the home during the first period of the pandemic, Bergmann and Wagner (2021) confirm smaller care networks during this period and indicate that one out of every five people had difficulty in obtaining adequate care from outside the household. And yet, the provision of personal care to parents outside one’s own household strongly increased across Europe (ibid). Generationally, strong shifts in informal care were observed from younger to older generations (mainly reflected in falls of parental and grandparental care). The findings confirm significant physical and mental health burdens associated with the increase in informal care (ibid).

“Privatization” is again here an over-arching trend. As mentioned, there was, for example, little additional public resourcing of care in either the long-term care or childcare domains but there was considerable private resourcing and indeed a return to family especially in the case of children. Parental leave changes in European Union countries and the United Kingdom are instructive.

In general, across Europe children’s vulnerability during COVID-19 was recognized in terms of resourcing their parents, either with paid time free from employment or extra financial support. In fact, this was one of the innovative social policy actions taken during the pandemic. Only four countries were inactive in regard to parental leave (Croatia, Hungary, Ireland and the United Kingdom), and in some of these there was only limited lockdown of childcare and education services, thereby diminishing the need for leave (Rubery and Tavora 2020 1). Across countries, the exigencies that triggered a parental leave policy response were either a child becoming ill with COVID-19 or a child/parent becoming unable to avail of a relevant education or care service through either exclusion of the child or the shutdown of the services.

The two main policy levers appear to have been parental leave and/or income support for parents out of work for childcare-related reasons. Table 1 groups 23 European countries^2^ on the basis of their main response. Nations are separated not just by their mode of response but also by the degree to which they instituted something new. Some countries could be said to have been “tidying up” either by ensuring legal regularity or coverage, whereas others appear to have seen themselves as coping with a new exigency (and therefore instituting a new provision).

**TABLE 1:** Countries Classified on the Basis of their Response to Parental Caring for Children in Response to the First Periods of COVID-19 in 2020.

A special and new paid parental leave	Austria, Belgium, France, Germany, Greece, Italy, Poland, Romania, Sweden
Modified existing sickness or other leave to include parental caring	Denmark, Estonia, Lithuania, Netherlands
No specific leave but new cash allowances for work absence	Bulgaria, Finland, Malta, Portugal, Slovenia
Employment protection, unpaid leave but no cash	Spain
No action to support parental caring	Croatia, Hungary, Ireland, United Kingdom

Source: Gentilini et al., 2020.

Nine countries introduced a new COVID-19 specific paid parental leave (Spain introduced it on an unpaid basis). While the constituent elements and attaching conditions varied, all of the measures were temporary but all targeted the needs of the child in terms of parental care. A further but closely related type of response is to be found in the four countries that modified existing leave schemes to take account of involuntary absence from work for child-care reasons. Denmark, Estonia, Lithuania and Netherlands changed either sickness leave or maternity provision or emergency leave to accommodate and support parental caring during the emergency periods of the pandemic. In another variation, countries eschewed introducing or amending leave but provided additional cash/income support to families where a parent had to be out of work to care for children. This resembles a form of unemployment compensation (rather than leave). This was the major line of action in five countries: Bulgaria, Finland, Malta, Portugal and Slovenia. These supports took different forms and varied in terms of duration and generosity but at root they constituted a recognition of the income support needs incurred by parents whose children needed their care while home-based.

What might have been done? From a gender perspective, the relatively sophisticated targeting in the existing set of parental leaves from a gender and work-life balance perspective could have informed the response. This might have seen leaves targeted at both mothers and fathers rather than the blanket measures that were adopted. Statistics are not yet available on take-up of the leave by gender but traditional gender patterns can be expected (and are in any case confirmed by evidence on differential time inputs by gender to informal work and care during the pandemic, e.g., Carers Week 2020; Rubery and Tavora 2020). Leave arrangements might also have been introduced for those with other caring responsibilities. We know that informal care of older, disabled or ill adults increased dramatically during the pandemic. In the United Kingdom for example, it has been estimated that the number of informal carers increased by 4.5 million during the first months of the pandemic in 2020 (out of an estimated total of 13.6 million informal carers in all) and that the pandemic saw some 2.8 million extra workers trying to fit in their care responsibilities around their employment (Carers Week 2020). This brought the number of United Kingdom workers with informal care responsibilities to nearly 7 million. One might have expected a wider recognition of the care support needs (financial and services) of such workers and indeed of informal carers in general. The current state of play in the EU is that of the 27 EU member states, 13 pay informal care allowances directly to informal care providers, 9 to the person needing care to spend on either formal or informal care and 5 have no payment system for informal care (Ecorys 2021). Although data is scarce, as far as is known no country introduced new benefits for informal carers and most oversaw a cutback in direct services and supports such as respite care.

### The Values

Did care become a normal subject of politics (as Sevenhuijsen 2003 saw as a trend some 20 years ago)? The answer is “yes and no”. Care was certainly more widely present in public discourse and public awareness and government discourse in some countries (Chatzidakis et al., 2020). The pandemic did open a window in which care was present, but it was located among many other considerations. As is obvious from the foregoing, care continued as largely private and personal but there were forces pulling in somewhat different directions. There are several ways in which this could be read from the evidence. First, the dominance of recourse to family endorses the conventional view of family as the institution of caring. Moreover, there was something of a reversion to a male breadwinner model (as epitomized in some of the measures on parental care-giving) with the blanket reliance on family concealing a reality of the main burden of care-giving falling on women (Fisher and Ryan 2021). However, and this is the second point, some politicization of care took place during the first height of the pandemic itself. Evidence of this from the United Kingdom, for example, saw Ministers having to speak of social care when they made public pronouncements about the pandemic (albeit only after the care home debacle was revealed by the media and following strong political interventions by care home owners and the families of those in care homes) (Daly 2020a). But this is at best a symbolic form of politicization unless it leads to a focused process of reform around the different elements I have considered throughout this article.

There is no evidence as yet of a sea change in the approach to and appreciation of care in national value systems. Carers are expected to care about and feel for those to whom they provide care, and as Meagher (2006: 35) says to draw on the moral bonds of the private sphere in their work. Moral and relational framings define the field of long-term (and other forms of) care to a degree that could be argued to be unique among workers or occupations at this level. One sees in some of the care policies, and to some extent also in public discourse, an idealization of care as approximating family love and commitment, emphasizing (emotional) attachment, personal investment and selflessness (Meagher 2006). A base complication is that paid care is never fully divorced from unpaid care and the extent to which it can be (or should be) commodified is continuously questioned (e.g., Radin 1996; Claassen 2011).

So it is not just structural issues that are at the root of the kinds of patterns that I have been uncovering here but also matters relating to values and culture.

## Conclusion

The analysis undertaken above allows us a first take at questions around the policy configuration during the 2020 phases of COVID-19 in regard to care for children and adults. The evidence confirms a patchy and varied response, both within, and across countries.

It is generally agreed that pre-existing structural and other challenges compounded the impact of the pandemic. In the long-term care sector for example, such factors include chronic and widespread underinvestment, lack of attention to safety and crisis preparedness and poor working conditions for many (Rocard et al., 2021). In the childcare sector, they include the development of out-of-home childcare under the rubric of reconciliation of work and family life and as a support for paid labour as against a necessary support for people who are genuinely dependent, a right for children and a social activity in its own right. There are real but somewhat different tensions in both. Early childhood education and care tends to be framed as a matter of social investment—investing in the capital of the younger/est generations—whereas long-term care is rarely if ever configured as a form of positive investment of public resources. Indeed, it is often hosted through negative imagery and rhetoric, portrayed as a problem field and located in a particular type of politics. Childcare is enmeshed in the politics of family support and employment policy whereas care for older people is politically a matter of welfare assistance and health policy (although the EU is trying now to frame it in terms of “work-family balance” (Spasova et al., 2018)).

In terms of thinking about the future, while I have tried throughout this piece to identify elements of a better response, in truth a major change of thinking is needed. The scale of the change involved calls for an overaching (rather than sectoral) view of care. Care as an overarching prism places the spotlight on the organization of care, the distribution of care-related responsibilities and the relationship between the productive and reproductive systems.

One strong reform vision is contained in the idea of *Universal Basic Services* (Coote and Percy 2020). This highlights the diversity of needs associated with care and emphasizes two principles: collective responsibilities, shared needs. Along with care (long-term and for children), these authors include health, education, transport, housing and information and communications as services that should be provided on a universal basis. Aspects of their proposal include a customized approach to meeting care-related needs, a variety of responses/services types and a better balance between top-down and bottom-up politics and resources. What a universal basic services approach means is that care is encompassed within a general vision that recognizes that all people need services and that access should be on the basis of need rather than ability to pay. The ethical case for looking after disabled, frail and vulnerable members of society is to the fore. These authors advocate providing services through a range of organizations, including co-ops and social enterprises and other forms of common ownership. In this and other ways, the role of public institutions is transformed as is the service landscape.

A second set of ideas centres on better acknowledging and rewarding care work in the *care economy*. There are different versions of this approach extending from recognizing care work as a form of market investment and profit and economic growth to greater public investment, regulation and job protection in the care sector. Nancy Folbre is a leading scholar here (see Folbre 2018) as is Himmelweit (2014). De Henau et al. (2016) conceptualize the care economy to include activities in education, care and health services and social care. In focusing on both the practice of care and its broader organization, an underlying mission is to place value on care work as an activity, to recognize it as skilled (rather than a “naturalized” female attribute) and to transform the paid care sector from a growing dependence on precarious working conditions for its workers into a sector of high-value. Concerned especially about the concealed and under-resourced nature of much of care provision and increasing marketization, such theorizing suggests the need to make the hidden extent and nature of care work visible and to do so in a way that is sensitive to larger trends and global changes. The United Kingdom based Women’s Budget Group, 2020 has also made a significant contribution to developing thinking on the concept, bringing together interpersonal, environmental and economic reform considerations to develop the notion of a caring economy (or an economy that cares) oriented towards gender equality, sustainability and wellbeing. All care workers—and indeed workers in general—are included in this vision with a renewed care economy setting a progressive path for all workers.

*Caring democracy*—as developed by Joan Tronto (2013)—is probably the broadest perspective of the three. Drawing from feminist care theory which emphasizes relationality and rejects the liberal notion of human beings as autonomous, Tronto argues that care rather than the market and economic growth should be at the centre of political life. She is critical of both the democratic deficit and the care deficit (the shortage of care workers) and, indeed, links them both, criticizing contemporary views of citizenship as being too narrow and pointing out how democracy depends on care-related and other activities that are often ignored. Vital issues around how we care and are allowed to care draw to the fore broad questions about power and the intersections of class, race, gender and other grounds for inequality in impoverishing contemporary citizenship. A caring democracy, for Tronto, centres on the care practices of democratic citizens, and offers a new vision for how the (re)distribution of care can improve democracy.

In summary, a basic problem is that we have not devised an equality respecting system to replace the full-time caretaking labour of women in the home. So we see a variety of halfway houses (one and a half breadwinner models and so forth) that are not supported by policy and that in any case place care as secondary. I agree with the scholars cited above that a fundamental discussion has to be held about how we value care, how we are going to recognize dependence and interdependence as normal and how we can treat care as a community and collective responsibility. There are real issues around social sustainability involved.

## Data Availability

The original contributions presented in the study are included in the article/Supplementary Material, further inquiries can be directed to the corresponding author.

## Footnotes

1 Along with caring for, care-giving and care-receiving.

2 These are the countries for which the information was most robust at the time of writing. Excluded from the EU member countries are: Cyprus, Czech Republic, Luxembourg, Latvia and Slovakia.
